# Optimizing Uptake of Long-Acting Injectable Pre-exposure Prophylaxis for HIV Prevention for Men Who Have Sex with Men

**DOI:** 10.1007/s10461-023-03986-5

**Published:** 2023-01-21

**Authors:** Lorraine T. Dean, Zachary Predmore, Alexandra Skinner, Siena Napoleon, Philip A. Chan, Julia Raifman

**Affiliations:** 1 Department of Epidemiology, Bloomberg School of Public Health, Johns Hopkins University, Baltimore, MD USA; 2 RAND Health Care, RAND Corporation, 20 Park Plaza, Suite 920, Boston, MA 02116 USA; 3 Department of Health Law, Policy & Management, Boston University School of Public Health, Boston University, Boston, MA USA; 4 Department of Medicine, Brown University, Providence, RI USA

**Keywords:** Preference elicitation, HIV prevention, Pre-exposure prophylaxis, LAI-PrEP, MSM, Implementation

## Abstract

Pre-exposure prophylaxis (PrEP) is a highly effective HIV prevention tool. Long-acting injectable PrEP (LAI-PrEP) offers another opportunity to reduce HIV. However, how at-risk individuals will consider LAI-PrEP over other modes of administration is unclear. We conducted a discrete choice experiment on preferences for PrEP among a sample of N = 688 gay, bisexual, and other men who have sex with men (GBMSM). We analyzed preferences for mode of administration, side-effects, monetary cost, and time cost using a conditional logit model and predicted preference for PrEP options. LAI-PrEP was preferred, despite mode of administration being the least important PrEP attribute. Side-effects were the most important attribute influencing preferences for PrEP (44% of decision); costs were second-most-important (35% of decision). PrEP with no side-effects was the most important preference, followed by monthly out-of-pocket costs of $0. Practitioners and policymakers looking to increase PrEP uptake should keep costs low, communicate clearly about PrEP side-effects, and allow the use of patient-preferred modes of PrEP administration, including LAI-PrEP.

## Resumen

La profilaxis prexposición (PrEP) es una herramienta de prevención del VIH muy eficaz. La PrEP inyectable de acción prolongada (LAI-PrEP) ofrece otra oportunidad para reducir el VIH. Sin embargo, no está claro cómo las personas en riesgo considerarán LAI-PrEP sobre otros modos de administración. Realizamos un experimento de elección discreta sobre las preferencias por la PrEP entre una muestra de N = 688 hombres homosexuales, bisexuales y otros hombres que tienen sexo con hombres (GBMSM). Analizamos las preferencias por el modo de administración, los efectos secundarios, el costo monetario y el costo del tiempo mediante un modelo logit condicional y la preferencia prevista por las opciones de PrEP. Se prefirió LAI-PrEP, a pesar de que el modo de administración es el atributo de PrEP menos importante. Los efectos secundarios fueron el atributo más importante que influyó en las preferencias por la PrEP (44% de la decisión); los costos fueron los segundos más importantes (35% de la decisión). La PrEP sin efectos secundarios fue la preferencia más importante, seguida de costos de bolsillo mensuales de $0. Los médicos y legisladores que buscan aumentar la aceptación de la PrEP deben mantener los costos bajos, comunicar claramente los efectos secundarios de la PrEP y permitir el uso de los modos de administración de la PrEP preferidos por los pacientes, incluido LAI-PrEP.

## Introduction

The HIV epidemic continues to disproportionately impact gay, bisexual, and other men who have sex with men (GBMSM) [1]. Pre-exposure prophylaxis (PrEP) offers an effective approach to prevent HIV infections among GBMSM and reduce incidence in this population. There are currently two oral forms of PrEP available including tenofovir disoproxil fumarate/emtricitabine (TDF/FTC), which is also known as Truvada and was approved by the FDA in 2012, and tenofovir alafenamide/emtricitabine (TAF/FTC), which is also known as Descovy and was approved by the FDA in 2019 [2]. In addition, the recent US Food and Drug Administration (FDA) approval of long-acting injectable PrEP (LAI-PrEP) offers another effective tool for reducing new infections of HIV [3]. Preferences for PrEP are especially important to consider for African American/Black and Hispanic/Latinx GBMSM who are at high risk of exposure to HIV [4, 5], whose HIV rates remain high [6] and who have not been sufficiently reached by oral PrEP [7].

There are several barriers to obtaining PrEP, and patients often make trade-offs in their decisions to use PrEP. Financial and time costs are prohibitive for many individuals, and previous studies have demonstrated that financial and time costs are consistently reported as the main barriers expressed by many individuals considering PrEP [8–12]. Despite most private and state Medicaid plans covering PrEP [13], and private health insurers increasingly expanding coverage for PrEP, cost-sharing associated with these insurance plans, including high out-of-pocket costs in the form of co-pays, coinsurance, and deductibles (i.e., people are still “underinsured”) remain a challenge [14, 15]. In our previous study among MSM in three US cities who were prescribed PrEP, co-pays and deductibles for medical services were a greater barrier to accessing PrEP than the cost and co-pays associated with the medication itself [12]. Even those with prescription drug coverage through insurance plans could pay more than $2000 per year in co-pays for PrEP and its associated laboratory testing [16]. Patients must also spend the time required to attend appointments and refill prescriptions, potentially having to miss work and lose income. These monetary and time costs are not the only consideration for patients taking PrEP as they may be making decisions based on other aspects of PrEP, including the potential side-effects of PrEP and the possibility for stigma around sexual or drug use behavior that increase risk for HIV infection.

As new modes of administration for PrEP emerge, real-world implementation questions arise of how patients will decide which PrEP modes to use, and what financial, cost, and other trade-offs they may be weighing in their decision. Introduction of LAI-PrEP raises many implementation questions about the likelihood of uptake compared to daily oral PrEP, on-demand PrEP (also known as 2-1-1 PrEP, in which two pills are taken at least two but not more than 24 hours before sex, another pill 24 hours after the first, and a final pill taken 24 hours after the second) and a subcutaneous PrEP implant [17]. Optimizing PrEP implementation and maximizing reductions in HIV incidence require an understanding of the decision-making process related to PrEP including newer LAI-PrEP formulations among at-risk populations.

In this study, we conducted a discrete choice experiment (DCE) among a sample of racially diverse at-risk GBMSM to determine preferences for LAI-PrEP and other formulations, with the goal of identifying optimal approaches for effective implementation.

## Methods

### Development of DCE

DCEs are a class of conjoint analyses where respondents make choices between at least two hypothetical alternatives that vary in several key attributes. By making a series of choices, the independent impact of each attribute on preferences can be calculated. This approach better approximates the complexity in the real-world process of health decision-making in which the choice between engaging a treatment or not depends on several factors, rather than just one element. Preference elicitation methods have been used previously to assess preferences for HIV testing and treatment in the US [18, 19].

Before completing the DCE, participants were shown the following text: “In this next section, you will choose between two different potential PrEP choices. You will be shown some information about these two choices—the options are the same except for the things that differ here, including being equally effective at preventing HIV. You should select the PrEP option you would prefer.” To develop our DCE, we used the checklist of best practices for DCE developed by a working group from the International Society for Pharmacoeconomics and Outcomes Research (ISPOR) [20]. We conducted interviews with 25 GBMSM seeking care at a sexually transmitted infection (STI) clinic in Rhode Island to develop a list of potential attributes for inclusion [21]. We narrowed this list to a set of four attributes based on this formative work and the expert opinion of the research team: cost, travel time, mode of administration, and side-effects. The full list of attributes and levels and their exact phrasing is found in the appendix [22]. We developed levels for the costs and travel time attributes based on the ranges provided by participants according to the amounts they would be willing to pay for PrEP and how far they would be willing to travel for an appointment. Levels for mode of administration and side-effects were derived by clinical recommendations and experience. The DCE was programmed as an online survey in Qualtrics, using a randomized design to ensure balance of all levels. Each participant was faced with eight different choice tasks where they chose between two PrEP options. The DCE was placed in the middle of a larger survey on attitudes towards PrEP and willingness to pay for PrEP medication and services.

### Fielding the DCE

We recruited GBMSM between May 2020 and October 2021 through electronic advertising on several social networking applications targeted to GBMSM (Scruff, Jack’d) as well as targeted advertisements on Facebook and Instagram. To be eligible for the study, respondents needed to be 18 years or older, have been assigned male at birth or currently identify as male, have been sexually active with at least one man in the last 12 months, be HIV-negative, speak English or Spanish, and live in New England (Massachusetts, Connecticut, Rhode Island, Maine, New Hampshire, and Vermont). The DCE was available in English or Spanish depending on participant preference. Participants were given one week from starting to complete the survey and were blocked from taking it if someone from the same IP address had already completed the survey. Participants who completed the survey and provided an email address were sent an electronic $25 gift card. We dropped suspected bots or fake responses from the survey if they had inconsistency in responses (e.g., respondents who reported having sex with a man during the screener but not later on in the survey) or an IP address located outside of New England.

### Analysis of Results

We calculated descriptive statistics (means, medians, standard deviations, ranges) of demographic variables. For the DCE, we used a conditional logit model with an Efron approximation to estimate preferences for attribute levels. We used dummy coding and set the least preferred level for each attribute to zero. We conducted a subgroup analysis of the coefficients based on several survey questions: participant race and ethnicity, participant self-reported income (above or below $75,000 per year), whether the participant reported ever taking PrEP in the past (even if just one pill), and whether the participant described themselves as willing to take PrEP in the future (definitely or probably willing, compared to those who were maybe, probably not, or definitely not willing). We calculated the relative importance of each attribute, or the percent of the decision that is associated with each attribute, by taking the distance between the highest and lowest coefficient within an attribute and normalizing across all attributes. We also simulated preferences for this population making hypothetical choices between different PrEP options. We predicted the share of respondents preferring a theoretical option by using the coefficients of the conditional logistic model. All analyses were conducted in R using the RStudio application (2021.09.02 Build 382) and the “clogit” command. The study was approved by the Institutional Review Board at Miriam Hospital in Providence, Rhode Island.

## Results

### Respondent Demographics

A total of N = 688 GBMSM participated in the study. Demographic characteristics and PrEP use history for this sample are found in Table 1. Most of the participants had never used PrEP but most were willing to use it. Some of the demographic variables do not sum to N = 688 because respondents did not answer or indicated they did not know the answer to certain questions.

**Table 1:** Demographics of Discrete Choice Experiment Respondents (N = 688, rows may not sum to 688 due to missing data)

Demographic	Mean or N (range or %)
Age	36.5 years (19–75)
Gender	
Cisgender male	668 (97.1%)
Transgender male	7 (1.0%)
Non-binary/gender non-conforming	5 (0.7%)
Race and ethnicity	
Hispanic/Latino	137 (19.9%)
Non-Hispanic Black	120 (17.4%)
Non-Hispanic Other Race	52 (7.6%)
Non-Hispanic White	379 (55.1%)
State of residence	
Rhode Island	462 (67.2%)
Massachusetts	153 (22.2%)
Connecticut	39 (5.7%)
Maine	22 (3.2%)
New Hampshire	9 (1.3%)
Vermont	3 (0.4%)
Educational attainment	
High school or less	16 (2.3%)
Some college	126 (18.4%)
Technical/vocational or associates degree	87 (12.7%)
Four-year college	216 (31.5%)
Graduate degree	240 (35.0%)
Health insurance status	
Private health insurance	501 (73.4%)
Public health insurance	129 (18.9%)
Uninsured	53 (7.8%)
Annual income	
$35,000 or less	108 (15.9%)
$35,001 to $50,000	146 (21.5%)
$50,001 to $75,000	172 (25.3%)
$75,001 to $100,000	88 (12.9%)
$100,001 or more	166 (24.4%)
Relationship status	
No current sexual partners	149 (21.8%)
Casual sexual partners	265 (38.8%)
Non-monogamous relationship	185 (27.1%)
Monogamous relationship	75 (11.0%)
Other	9 (1.3%)
Ever taken PrEP	
Yes	274 (40.3%)
No	406 (59.7%)
Currently taking PrEP	
Yes	206 (30.3%)
No	474 (69.7%)
Willing to take PrEP	
Definitely	254 (37.0%)
Probably	174 (25.3%)
Maybe	224 (32.6%)
Probably not	25 (3.6%)
Definitely not	10 (1.5%)

### Importance of PrEP Attributes

**Fig. 1:**
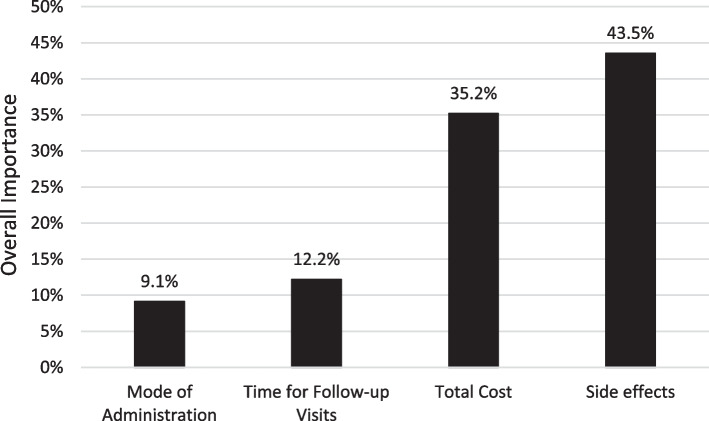
Relative Importance of Each Included Attribute (Percent to which the attribute contributed to the decision to use PrEP)

Figure 1 shows the importance of each attribute for the decision between PrEP options overall. Mode of administration (9.1%) was the least important attribute. The two most important PrEP attributes were side-effects (43.5%) and total out-of-pocket cost (35.2%), followed by time for follow-up visits (12.2%).

### Preferences for Each Level Within Each Attribute

Table 2 shows the conditional logit model coefficient for each level within each attribute. These coefficients are the results of a logistic regression that shows how the presence of each level within a PrEP option affected the participant’s choice. Higher coefficients indicate that participants were more likely to choose a PrEP option with that level, while lower coefficient values are associated with being less likely to choose PrEP. Coefficients for an attribute should be interpreted relative to those of other attributes within that level and values are interpreted in comparison to other attribute values in the DCE. The coefficients for $200, long term side-effects, 4-h travel time, and a PrEP implant were the lowest. Among modes of administration, individuals were most likely to prefer injection every few months (coefficient 0.16, 95% CI 0.09, 0.23), followed by a pill at the time of sex (coefficient 0.08, 95% CI 0.00, 0.15), then a daily pill (coefficient 0.01, 95% CI -0.07, 0.09), and an implant every few months (coefficient constrained to be 0). However, costs and side-effects exhibited the strongest influences on PrEP preferences, over and above mode of administration. Lower monetary cost PrEP had higher logistic coefficients. PrEP with an out-of-pocket cost of $0 was the most preferred, with a coefficient of 0.62 (95% CI 0.52, 0.71), followed by $10 PrEP at 0.61 (95% CI 0.51, 0.70), $25 PrEP at 0.41 (95% CI 0.31, 0.51), $50 PrEP at 0.40 (95% CI 0.30, 0.50), $100 PrEP at 0.26 (95% CI 0.16, 0.36), and $200 PrEP was the reference value with a coefficient of zero. PrEP with no side-effects was the most preferred with a coefficient of 0.76 (95% CI 0.69, 0.84), side-effects upon starting was 0.55 (95% CI 0.47, 0.63), and side-effects that persist while on PrEP were 0.42 (95% CI 0.25, 0.42) with long-term side-effects as the reference value. Shorter travel times were also generally associated with higher coefficients, with 30-min travel time having a coefficient of 0.21 (95% CI 0.13, 0.30), one hour 0.19 (95% CI 0.10, 0.27), two hours 0.16 (95% CI 0.07, 0.25), and three hours 0.17 (95% CI 0.09, 0.26) with four hours as the reference value.

**Table 2:** Logistic coefficients for each PrEP attribute

Attribute	Level	Coefficient	SE	Z-score	p-value
Side effects	No side effects	0.76	0.040	12.19	< 0.001
	Side effects on starting	0.55	0.041	13.44	< 0.001
	Side effects that persist	0.34	0.042	7.95	< 0.001
	Longer-term side effects	Constrained to be 0			
Total cost	$0	0.62	0.049	12.61	< 0.001
	$10	0.61	0.049	12.36	< 0.001
	$25	0.41	0.051	8.14	< 0.001
	$50	0.40	0.051	7.78	< 0.001
	$100	0.26	0.052	5.03	< 0.001
	$200	Constrained to be 0			
Time for Follow-up visits	30 min	0.21	0.043	4.93	< 0.001
	1 h	0.19	0.044	4.25	< 0.001
	2 h	0.16	0.044	3.66	< 0.001
	3 h	0.17	0.044	4.00	< 0.001
	4 h	Constrained to be 0			
Mode of administration	Daily pill	0.01	0.039	0.29	0.773
	Pill at the time you have sex	0.08	0.039	2.04	0.041
	Injection	0.16	0.038	4.21	< 0.001
	Implant	Constrained to be 0			

Table 2 shows these coefficients for all levels of attributes and the significance of each coefficient. Coefficients can be compared across attributes to show the relative preferences for each level. Cost and side-effects were the two most important attributes; specifically, “no side-effects” was the single most preferred PrEP level and $0 out-of-pocket cost was the second most important.

We also conducted an analysis to determine the coefficients associated with each attribute level for different demographic groups and those with different experiences with PrEP, with the results of this analysis found in the appendix [22]. Overall, lower income people (those making less than $75,000 per year) had statistically significantly higher coefficients associated with lower cost PrEP and lower coefficients associated with higher cost PrEP. Side-effects were more important for White respondents than for other racial and ethnic groups. Finally, we explored the coefficients associated with each level based on experience with and self-reported willingness to take PrEP in the future. Those who had taken PrEP in the past were more sensitive to out-of-pocket costs (relatively higher coefficients associated with lower costs) than those who had never taken PrEP. Those who were more willing to take PrEP in the future (probably or definitely willing) had higher coefficients associated with less side-effects and lower coefficients associated with worse side effects.

### Predicted Preference Shares

**Fig. 2:**
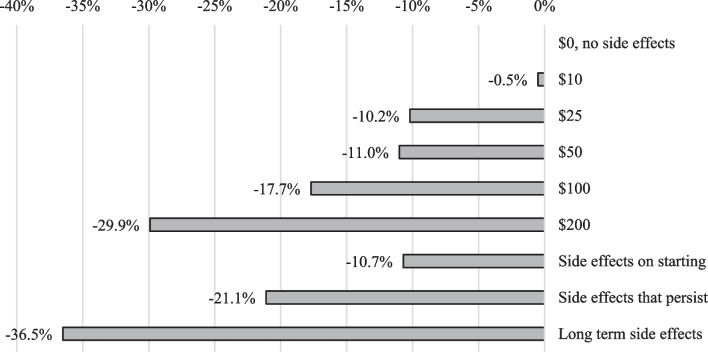
Effects of changing the characteristics on the average probability of uptake for PrEP for the overall survey population

Using the results of the DCE, we simulated preferences to predict how cost and side-effects (the most important attributes for preferences) influence the average probability of PrEP uptake in Fig. 2, starting from a 50% baseline. For example, if PrEP costs increased from $0 to $10, 0.5% of respondents would not be interested. Side-effects also made people less interested in PrEP; if PrEP had only side-effects on starting, 10.7% fewer people would be interested in PrEP compared to PrEP with no side-effects. The overall most preferred combination of attributes was PrEP that cost $0 out-of-pocket per month, 30 min travel time, had no side-effects, and was administered by injection every few months.

## Discussion

This is among the first studies to evaluate the decision-making process between different formulation of PrEP including LAI-PrEP among at-risk GBMSM. In this study of racially diverse GBMSM, we identified a strong preference for PrEP when offered as an injectable treatment, with no side effects, at no-cost, and with visit times of 30-min or less. LAI-PrEP was the most preferred mode of administration though more strongly preferred by White respondents than those of other races, by people who had taken PrEP in the past, and by people who had not previously used PrEP but expressed willingness to try it. Consistent with other studies of the impact of attributes of PrEP and their impact on preferences, lower out-of-pocket cost ($0) PrEP with no side-effects was the most preferred option to optimize PrEP use. These results can help guide PrEP implementation efforts and policy decisions to maximize PrEP uptake and effectiveness in reducing HIV incidence among at-risk populations.

Our study builds on evidence from previous DCEs on PrEP preferences. One DCE that recruited participants via gay social networking applications also found cost was the most important attribute, though side-effects were not included as an attribute. Given the importance of side effects in our study, this study characterizes the relationship and impact of both cost and side-effects on PrEP preferences [23]. In another DCE study of preferences for LAI-PrEP in a national sample of MSM, side-effects and cost were the two most important attributes [24], consistent with our results; this study expands on that work and shows the importance of these attributes across all modalities of PrEP. A DCE of PrEP preferences in the US military found mode of administration to be the most important attribute in both those with and without experience taking PrEP, but neither cost nor side-effects were included in that DCE [25]. Our results strongly suggest that keeping out-of-pocket costs and side-effects low increases interest in PrEP, regardless of mode of administration. The introduction of a new PrEP mode alone may not increase PrEP uptake without accompanying strategies to ensure out-of-pocket costs and side effects are low. Efforts to develop new formulations of PrEP may increase interest in at-risk populations but are likely not as important as efforts to keep PrEP out-of-pocket costs low, though some research suggests that when directly asked about preferences for LAI-PrEP, relatively few GBMSM say they would prefer that to oral PrEP [26]. Similarly, while time was not a major driver of preferences, respondents showed a clear preference for shorter travel times. Keeping time costs low through short visits and injections every few months could increase interest in and uptake of PrEP.

PrEP preferences also varied by demographic group and with different levels of PrEP experience. Lower-income respondents (< $75,000 per year in our sample) were more sensitive to out-of-pocket PrEP costs. Additionally, those with experience taking PrEP were more sensitive to these costs, suggesting that past experience with PrEP was negatively impacted by the actual out-of-pocket costs of PrEP they experienced. If health care providers, payers, and policymakers want to target PrEP to people with low incomes or those who may have taken PrEP in the past, out-of-pocket costs need to be kept low.

In our study, small increases in cost (e.g., from $0 to $10) were not associated with large decreases in interest in PrEP. The tradeoffs we simulated between cost and side-effects also suggests that low PrEP costs alone may not be enough to encourage uptake. Simulated PrEP uptake was more impacted by even short-term side effects upon starting than costs of PrEP of at least $25. Costs of $50 per month were needed to cause respondents to be less interested in PrEP with no side-effects than PrEP with side-effects only on starting. Respondents also had very strong preferences against long-term side-effects. Individuals would prefer paying $200 for PrEP with no side-effects than pay nothing for PrEP with long-term side-effects. This suggests that efforts to reduce PrEP out-of-pocket costs, in isolation of the other tradeoffs patients consider, may not be effective to increase interest in PrEP. Surveys have shown that many young GBMSM are not even aware of PrEP [27], so targeted messaging efforts by public health officials about the safety and efficacy of PrEP may be the best way to increase PrEP uptake among high-risk youth and others unfamiliar with PrEP, especially given the importance of low out-of-pocket costs for PrEP among young GBMSM [28]. Among those not reporting interest in PrEP, there was no single attribute that stood out as being most important to increase interest in PrEP among that group.

Keeping out-of-pocket costs low or none is of critical importance to maximize uptake. The US has several models for PrEP assistance that can make total out-pocket costs close to $0. In addition to the manufacturer’s coupon programs and other PrEP assistance programs, federal guidelines as of 2021 mandated that non-grandfathered Affordable Care Act compliant private health insurance plans cover services associated with PrEP, including provider visits, HIV and STI testing to remain eligible monitoring of kidney function, and others [29]. These recent decisions are a step in the direction of realizing the ideal combination of PrEP attributes that can increase uptake. However, covering out-of-pocket costs for uninsured individuals and especially in states that have not expanded Medicaid is still major challenge.

### Limitations

DCE is a stated preference methodology; though we tried to keep choices simple and similar to those that may be encountered in real-world settings, respondents may not be familiar with making these decisions. For example, at the time of the survey, implant and LAI-PrEP were not approved PrEP modalities in the US. However, including these as options gave us the opportunity to assess emerging technologies and compare those to the current standard of care. In the interest of lowering cognitive burden for respondents, we also only presented a subset of the available attributes that people may consider when making a decision about PrEP. We only had respondents complete eight choice tasks with four attributes in order to increase survey completion rates; this is on the lower end but still within standard DCE practice [30]. While the results of this DCE show the relative importance of each of these attributes, the specific numeric values of the coefficients presented are dependent on the attributes and levels used in this experiment. With different attributes or levels, these numeric values would likely be different, though their relative impact would remain consistent. Additionally, this sample may be unrepresentative of all US GBMSM. We limited our sample to those using gay social networking sites in New England, and most of our respondents were cisgender non-Hispanic White men. Future research on PrEP preferences could include more transgender or non-binary individuals as well. Doing an online survey could be biased towards those GBMSM with higher levels of education [31]. We used a set of checks and questions to flag potential bots or spammers, and like other surveys dropped a high percentage of respondents from outside of our geographic study area. [32]

### Implications

Despite these limitations, the results of this experiment highlight the complex decisions that GBMSM make when considering whether to take PrEP and which formulations. Policymakers should use these results to better develop strategies to increase the uptake of PrEP among MSM and prevent future HIV infections. Clear communication by the Centers for Disease Control and Prevention about the short- and long-term side-effects as well as the constant monitoring of side effects by physicians could help reduce the impact of fear of those side effects on PrEP uptake. Costs are also a barrier that can be addressed through policy. The US Preventative Services Task Force (USPSTF) recommendation on PrEP as an effective tool for HIV prevention (and associated “A” rating) should require most health plans in the US to cover PrEP medications without cost-sharing [33]. However, PrEP costs are complex and consist of more than just the cost of the medication, with lab testing and outpatient visits potentially adding up to hundreds or thousands of dollars in cost per year. Given the importance of cost in the decision to use PrEP, copay assistance programs through manufacturers and state policy efforts to reduce costs of PrEP could target these additional out-of-pocket costs to keep the overall costs of PrEP under $25 per month. Providers should be mindful as new, preferred modes of PrEP, like injections, are approved by the FDA. However, offering a new mode of administration may not substantially increase the appeal of PrEP on its own. Telemedicine and other virtual care approaches that have increased in use during the COVID-19 pandemic could be useful for reducing travel times for routine PrEP outpatient visits.

## Conclusion

In this DCE measuring PrEP preferences in GBMSM, PrEP delivered through injection every few months, with no side-effects, that cost $0 out-of-pocket per month, and had 30 min travel time, represented the most desirable package for PrEP. While LAI-PrEP was the most preferred mode of administration, mode of administration did not emerge as a strong driver of preferences for PrEP. Instead, side-effects and monetary cost were the two most important attributes predicting PrEP preferences. As PrEP is a key piece of the Ending the HIV Epidemic (EtHE) plan, efforts to scale up the use of PrEP are unlikely to succeed unless cost and side-effect barriers can be sufficiently addressed by health care providers and policymakers.

## Data Availability

Data is available from authors upon reasonable request.

## Appendix

See Tables

**Table 3:** Attributes and levels used in the discrete choice experiment

Attribute	Levels
Total cost (average per month)	• $0 • $10 • $25 • $50 • $100 • $200
Time for follow-up visits (travel time and clinic time, every 3 months)	• 30 min • 1 h • 2 h • 3 h • 4 h
Side effects	• No side effects • Some side effects on starting (headaches, nausea) then none • Some side effects on starting (headaches, nausea) that persist while you are on PrEP • Some longer-term side effects (bone and kidney problems) in later years on PrEP
Mode of administration	• Daily pill • Pill only around the time you have sex • Injection every few months Small implant every few months

3 and

**Table 4:** Logistic coefficients for different groups of respondents (mean (SE))

	High income (n = 254)		Low income (n = 426)		Hispanic (n = 137)		White (n = 379)		Black (n = 120)		Other (n = 52)		Not taken PrEP (n = 406)		Taken PrEP (n = 274)		Not willing PrEP (n = 259)		Willing to take PrEP (n = 428)	
Out-of-pocket cost																				
$0	0.48 (0.08)		0.71 (0.06)		0.45 (0.11)		0.65 (0.07)		0.69 (0.12)		0.68 (0.17)		0.49 (0.06)		0.79 (0.08)		0.57 (0.08)		0.65 (0.06)	
$10		0.57 (0.08)		0.63 (0.06)		0.54 (0.11)		0.62 (0.07)		0.69 (0.12)		0.47 (0.17)		0.51 (0.06)		0.74 (0.08)		0.46 (0.08)		0.70 (0.06)
$25	0.35 (0.08)		0.45 (0.06)		0.39 (0.11)		0.46 (0.07)		0.40 (0.12)		0.09 (0.19)		0.31 (0.06)		0.57 (0.08)		0.37 (0.08)		0.45 (0.07)	
$50	0.44 (0.08)		0.38 (0.07)		0.35 (0.11)		0.47 (0.07)		0.42 (0.12)		-0.07 (0.19)		0.30 (0.06)		0.56 (0.08)		0.24 (0.08)		0.50 (0.07)	
$100	0.24 (0.08)		0.26 (0.07)		0.25 (0.11)		0.28 (0.07)		0.26 (0.13)		0.16 (0.18)		0.19 (0.07)		0.35 (0.08)		0.21 (0.08)		0.29 (0.07)	
$200																				
Time costs																				
30 min	0.18 (0.07)		0.23 (0.05)		0.16 (0.10)		0.33 (0.06)		0.05 (0.10)		− 0.08 (0.16)		0.24 (0.06)		0.19 (0.07)		0.15 (0.07)		0.25 (0.05)	
1 h	0.11 (0.07)		0.21 (0.06)		0.06 (0.10)		0.27 (0.06)		0.10 (0.10)		0.14 (0.15)		0.17 (0.06)		0.22 (0.07)		0.12 (0.07)		0.22 (0.06)	
2 h	0.18 (0.07)		0.15 (0.06)		0.11 (0.10)		0.26 (0.06)		0.01 (0.10)		− 0.11 (0.16)		0.17 (0.06)		0.16 (0.07)		0.19 (0.07)		0.14 (0.06)	
3 h	0.22 (0.07)		0.13 (0.06)		0.10 (0.10)		0.25 (0.06)		0.15 (0.10)		− 0.12 (0.16)		0.17 (0.06)		0.18 (0.07)		0.16 (0.07)		0.18 (0.06)	
4 h																				
Side effects																				
None	0.87 (0.07)		0.69 (0.05)		0.38 (0.09)		0.94 (0.06)		0.70 (0.09)		0.76 (0.15)		0.79 (0.05)		0.76 (0.06)		0.44 (0.06)		0.96 (0.05)	
Only upon starting	0.56 (0.07)		0.54 (0.05)		0.31 (0.09)		0.72 (0.06)		0.28 (0.10)		0.70 (0.15)		0.60 (0.05)		0.52 (0.06)		0.34 (0.06)		0.69 (0.05)	
Persistent	0.37 (0.07)		0.31 (0.05)		0.11 (0.09)		0.46 (0.06)		0.24 (0.10)		0.37 (0.16)		0.38 (0.06)		0.31 (0.07)		0.22 (0.07)		0.41 (0.06)	
Only long-term																				
Mode of administration																				
Daily pill	0.07 (0.06)		− 0.01 (0.05)		0.07 (0.09)		0.00 (0.05)		− 0.01 (0.09)		− 0.01 (0.15)		− 0.01 (0.05)		0.03 (0.06)		− 0.08 (0.06)		0.07 (0.05)	
Pill at the time of sex	0.05 (0.06)		0.10 (0.05)		0.13 (0.09)		0.08 (0.05)		− 0.01 (0.09)		0.12 (0.14)		0.08 (0.05)		0.08 (0.06)		0.05 (0.06)		0.10 (0.05)	
Injection	0.13 (0.06)		0.18 (0.05)		0.25 (0.09)		0.18 (0.05)		− 0.03 (0.09)		0.20 (0.14)		0.11 (0.05)		0.22 (0.06)		0.08 (0.06)		0.21 (0.05)	
Implant																				

4.

## Code Availability (Software Application or Custom Code)

All analyses were conducted in R, using the RStudio application (2021.09.02 Build 382).
